# The Influence of Laboratory Scanner versus Intra-Oral Scanner on Determining Axes and Distances between Three Implants in a Straight Line by Using Two Different Intraoral Scan Bodies: A Pilot In Vitro Study

**DOI:** 10.3390/jcm12206644

**Published:** 2023-10-20

**Authors:** Asaf Shely, Diva Lugassy, Ophir Rosner, Eran Zanziper, Joseph Nissan, Shir Rachmiel, Yara Khoury, Gil Ben-Izhack

**Affiliations:** 1 Department of Oral Rehabilitation, The Maurice and Gabriela Goldschleger School of Dental Medicine, Sackler Faculty of Medicine, Tel Aviv University, Tel Aviv 6997801, Israel; asafshely@gmail.com (A.S.); rosnerop@yahoo.com (O.R.); eranzen@gmail.com (E.Z.); nissandr@gmail.com (J.N.); shir.rachmiel@gmail.com (S.R.); yarakhoury@mail.tau.ac.il (Y.K.); 2 Department of Orthodontics, The Maurice and Gabriela Goldschleger School of Dental Medicine, Sackler Faculty of Medicine, Tel Aviv University, Tel Aviv 6997801, Israel; diva.lugassy@gmail.com

**Keywords:** CAD-CAM, scan abutment, ISB, implant axis, laboratory scanner, intra-oral scanner, CEREC, Primescan, dental implants, oral rehabilitation

## Abstract

Background: The purpose of this in vitro study was to compare the inter-implant distance, inter-implant axis, and intra-implant axis of three implants in a straight line by using a laboratory scanner (LBS) versus an intra-oral scanner (IOS) with two different intra-oral scan bodies (ISBs). Methods: A 3D model was printed with internal hex implant analogs of three implants in positions 15#, 16#, and 17#. Two standard intra-oral scan bodies (ISBs) were used: MIS ISB (two-piece titanium) and Zirkonzhan ISB (two-piece titanium). Both ISBs were scanned using 7 Series dental wings (LBS) and 30 times using Primescan (IOS). For each scan, a stereolithography (STL) file was created and a comparison between all the scans was performed through superimposition of the STL files by using 3D analysis software (PolyWorks^®^ 2020; InnovMetric, Québec, QC, Canada). A Kolmogorov–Smirnov test was performed followed by a Mann–Whitney test (*p* < 0.05). Results: The change in inter-implant distance for the MIS ISB was significantly lower compared to the ZZ (*p* < 0.05). The change in intra-implant angle was significantly lower for the ZZ ISB compared to MIS (*p* < 0.05). The changes in inter-implant angle between the mesial and middle and between the middle and distal were significantly lower for MIS compared to ZZ in contrast to mesial to distal, which was significantly higher (*p* < 0.05). Conclusions: Both ISBs showed differences in all the parameters between the LBS and the IOS. The geometry of the scan abutment had an impact on the inter-implant distance as the changes in the inter-implant distance were significantly lower for the MIS ISB. The changes in the intra-implant angle were significantly lower for the ZZ ISB. There is a need for further research examining the influence of geometry, material, and scan abutment parts on the trueness.

## 1. Introduction

The digital era that we experience today gave us the technology for scanning both implants and teeth. Scanners (intra-oral and extra-oral) have many advantages such as improved patient comfort, reduced distortion from impression materials and plasters, reduced chair time, passive impressions, and the acquisition of digital files [1,2,3,4]. Two methods can be used for a digital impression: indirect (laboratory scanner), where the technician may scan either a model or impression, or direct (intra-oral scanner), where the dentist uses a chair-side scanner [5]. Today, the traditional method is still in use and many dentists take conventional impressions (polyether, vinyl polysiloxane, and vinyl polyether siloxane) [6], though there are many studies that demonstrate that when using full digital protocols for a full-arch-implant-supported prosthesis, we achieve a good accuracy compared to conventional methods [7,8,9,10,11].

When taking a three-dimensional scan, we receive an STL (stereolithography) file. We can design works on the CAD system as the STL file is an algorithm of a CAD mesh, which transmits the three-dimensional scan with a high degree of accuracy, and the accuracy of the file is determined by the trueness and precision of the scan. Trueness refers to how close the measurements are to the reference or actual dimension of the object being scanned, while precision refers to the consistency of the scans [12,13]. When scanning implants, we need to use intraoral scan bodies (ISBs), which have a variety of compositions, the most common of which are titanium type 5, polyether ether ketone (PEEK), or a combination of both. The ISB can be one piece or two pieces [14]. There are different geometries for ISBs, and they differ between commercial companies. The geometry is important for receiving an accurate position and orientation of the implants. The diameter and position of the ISB affect the trueness of implant impressions. After receiving the data from the scan, the software analyzes the data and defines the implants’ longitudinal, vertical, and rotational axes [15,16]. Revilla-León et al. reported that the optimal ISB design may vary depending on the intraoral scanner (IOS), the materials (PEEK or titanium), the implant angulation, the inter-implant distance, the implant depth, and other factors [17]. Today, laboratory scanners serve as the gold standard when compared to intra-oral scanners because of their higher degree of accuracy [18,19].

Regarding digital files, there are several terms: value—a quantitative characterization; trueness—the proximity of the test results or measurements to the true value; precision—the proximity between the test results; and accuracy: the test results should be close to the true value and to each other (trueness and precision together) [20,21].

Capturing the implant axes is clinically significant as it determines the accuracy of the prosthesis and the passive fit and affects the design and structure of the final restoration, for example, occlusal contacts, contact areas, and passive fit. The ongoing improvement in the digital impression techniques and in the ISB design allows us to fabricate prostheses that are more accurate, and the passive fit between cemented and screw-retained crowns has become an insignificant factor [22,23].

The purpose of this study was to measure the changes (trueness) in the position (intra-implant distance, inter-implant distance, intra-implant angle, and inter-implant angle) of three implants in a straight line (positions #15, #16, #17) by comparing laboratory scanning (LBS, gold standard) to intra-oral scanning (IOS) with two different ISBs. Our null hypotheses were that no difference will be found between the intra-oral scanner and laboratory scanner, and that no difference will be found between the two different ISBs.

## 2. Materials and Methods

**Figure 1:**
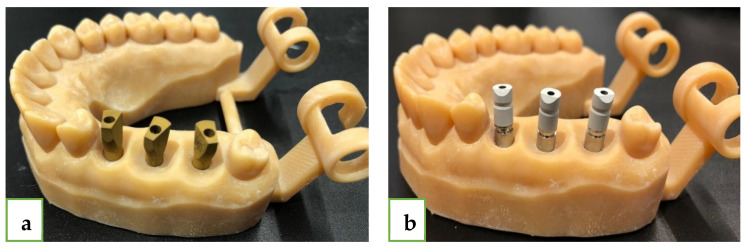
(**a**) Three MIS ISBs in place of teeth 15, 16, 17. (**b**) Three ZZ ISBs in place of teeth 15, 16, 17.

In this research, we printed a resin V-Print model by using a SolFlex 650 × 350 3D printer (VOCO GmbH, Heidelberg, Germany). In place of teeth #15 (1), #16 (2), #17 (3), we used an MIS standard internal hexago.0n22 implant analog, diameter 3.75 mm and length 11.5 mm. Two types of ISB were used (Figure 1):

1. MIS ISB (MIS, Titanium, two piece), asymmetrical geometry, internal hex connection.
2. Zirkonzahn ISB (ZZ, Titanium, two piece), cylindrical/asymmetric geometry, internal hex connection.

MISISB and ZZ ISB were screwed to the analogs by using electronic implant prosthetic screwdriver iSD900 (NSK^®^, Osaka, Japan) at 20 N·m. For creating the reference model, both ISBs were scanned using 7 Series dental wings (Dental wings^®^, Montreal, QC, Canada) laboratory desktop scanner. We produced a QR file and then the file was converted to STL file. In terms of accuracy, this scanner and the receiving scan are considered the gold standard [19]. The intra-oral scans (in vitro) were performed using a Primescan (CEREC^®^ Primescan; Dentsply Sirona, Milford, DE, USA) scanner and the protocol for scanning that is suggested by Sirona was used. Both MIS ISB and ZZ ISB were scanned thirty times, receiving thirty STL files, one for each scan. The comparison between each of the thirty scans to the laboratory scan (reference model) was performed using digital software (PolyWorks^®^ 2020; InnovMetric, Québec, QC, Canada) using the best-fit method.

**Figure 2:**
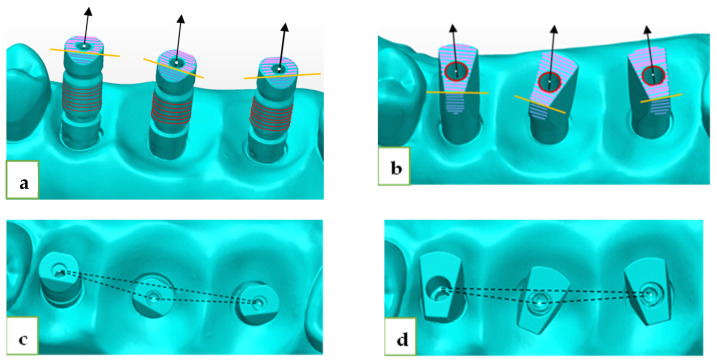
(**a**) ZZ upper plane, cylinder, axis, central point, side plane, and sideline. (**b**) MIS upper plane, cylinder, axis, central point, side plane, and sideline. (**c**) ZZ inter-implant distance. (**d**) MIS inter-implant distance.

Both ISBs (ZZ and MIS) had different geometries, yet both had a flat surface that was pointed to the buccal (Figure 1). The mesial abutment was defined as number 1 (#15), the medial as number 2 (#16), and the distal as number 3 (#17). Because of the special geometry of both MIS ISB and ZZ ISB, we were able to compare these two ISBs. A superimposition (best-fit algorithm) of the LBS scan with each of the IOS scans (thirty times for each ISB) was performed by using PolyWorks Inspector™ 2020 Software Verification and Measurement. The superimposition was also based on the adjacent teeth of the model. By performing the superimposition process, we were able to measure all the axes and distances that we defined. Subsequently, we were able to calculate the spatial characterization of each ISB relative to the reference model (axes and distances). We used several definitions (Figure 2):

- Upper plane (pink plane)—defined as the top surface of the ISB.
- Cylinder (red line)—defined as the associated best-fit inner cylinder of MIS ISB and outer cylinder of ZZ ISB.
- Axis (black line)—defined by the longitudinal axis of the associated best-fit cylinder to the ISB.
- Central point (white dot)—defined by the point of intersection between the cylinder and the upper plane of the ISB.
- Side plane (blue plane)—defined as the associated best-fit side plane of the ISB.
- Side line (yellow line)—defined by the line of intersection between the upper plane and the side plane, used for defining the system of axes of each ISB.
- Inter-implant distance (black intermitted line)—defined as the distance between two central points: distance 1–2, distance 2–3, and distance 1–3. The deviation in each distance from the reference was calculated through subtraction.

**Figure 3:**
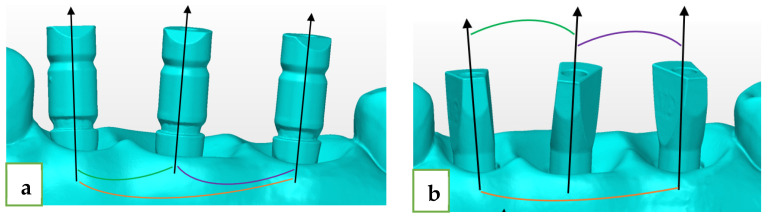
(**a**) ZZ intra-implant angle and inter-implant angle (delta axes). (**b**) MIS intra-implant angle and inter-implant angle (delta axes).

The angle formed between the axes of each of the two ISBs is defined as Delta axis 1–2, 2–3, and 1–3, (Figure 3):

- Delta axis 1–2 (green)—defined as the angle formed between the axis of the mesial and middle scan abutments.
- Delta axis 2–3 (purple)—defined as the angle formed between the axis of the distal and middle scan abutments.
- Delta axis 1–3 (orange)—defined as the angle formed between the axis of the mesial and distal scan abutments.

The deviation in each one from the reference model was calculated through subtraction.

**Figure 4:**
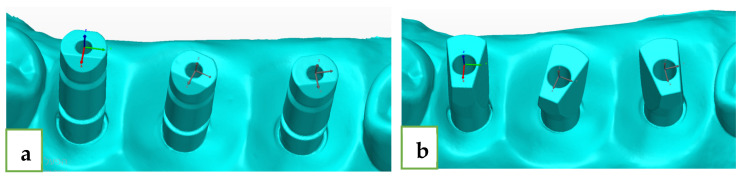
(**a**) ZZ x, y, z axes. (**b**) MIS x, y, z axes.

For every ISB of the reference model, we defined the axes, and the center of these axes was at the center of the upper plane. Buccal–lingual plane was defined as the X-axis (red) and the positive inclination was buccal. Mesial–distal plane was defined as the Y-axis (green) and the positive inclination was distal. Occlusal–gingival plane was defined as the Z-axis (blue) and the positive inclination was occlusal (Figure 4).

Superimposition was performed by using PolyWorks|Inspector™ 2020 Software Verification and Measurement. Both STL files from the LBS and from the IOS were superimposed (best-fit algorithm). Subsequently, we were able to measure the axes and distances of both the MIS ISB and ZZ ISB, and we calculated the spatial characterization of each ISB relative to the reference model as follows:

The distance between each central point of the LBS scan and its counterpart central point of the IOS scan was defined as the shift in the ISB head (both MIS and ZZ) with respect to the indirect scan from all the axes (white dot). This was calculated as follows:

D central point _1,2,3_ = $\sqrt{X^{2}+Y^{2}+Z^{2}}$

Delta axis _1,2,3_—calculated as the three-dimensional angle between each longitudinal axis of the LBS scan and its counterpart longitudinal axis of the IOS scan.

Intra-implant distance (mm)—the 3D changes between the D central points in each single ISB.

Inter-implant distance (mm)—the 3D changes between the D central points of two ISBs.

Intra-implant angle (angle)—the 3D changes between the delta-axis in each single ISB.

Inter-implant angle (angle)—the 3D changes between the delta-axis of two ISBs.

We performed a statistical analysis by using the Statistical Package for Social Sciences for Windows Release 23.0 (SPSS Inc., Chicago, IL, USA). A Kolmogorov–Smirnov test was used for independent variables that are not normally distributed. Mann–Whitney test was used for comparing within each group (ZZ and MIS). The statistical significance level for this work was *p* < 0.05.

## 3. Results

The Kolmogorov–Smirnov test that was performed on the study variables indicated a normal distribution (*p* < 0.05).

Table 1 shows the mean error, standard deviation (SD), range, and percentiles (P25, P50, P75) of the inter-implant distance, intra-implant distance (central points 1, 2, 3), intra-implant axes (delta axes 1, 2, 3), and inter-implant axes (12, 23, 13) for both ISBs.

Mann–Whitney tests were used to examine the differences between MIS and ZZ when comparing the IOS to the LBS (reference model).

**Figure 5:**
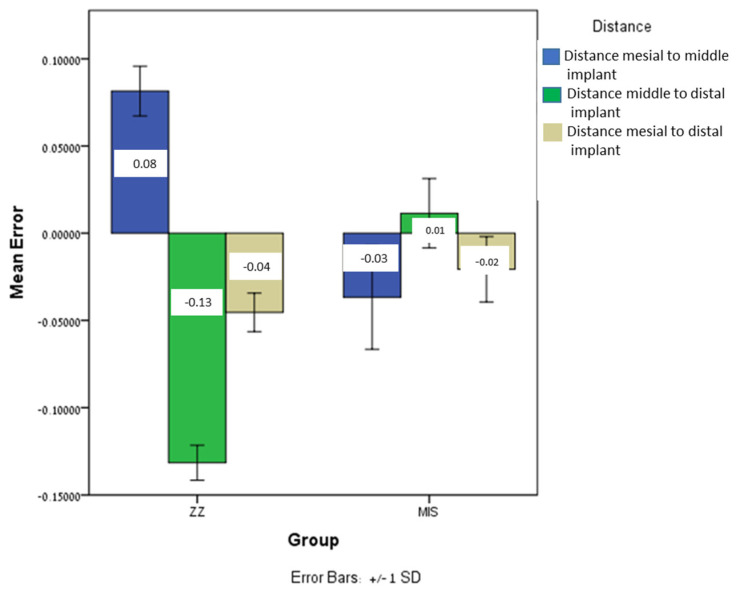
The mean inter-implant distance error and ±SD for both ZZ and MIS.

Significant differences were found between MIS and ZZ regarding the inter-implant distance: distance between mesial and middle implant (*p* = 0.0001), distance between middle and distal implant (*p* = 0.0001), and distance between mesial and distal implant (*p* = 0.0001). In all those variables, the mean error for MIS was lower than that for ZZ (Figure 5).

**Figure 6:**
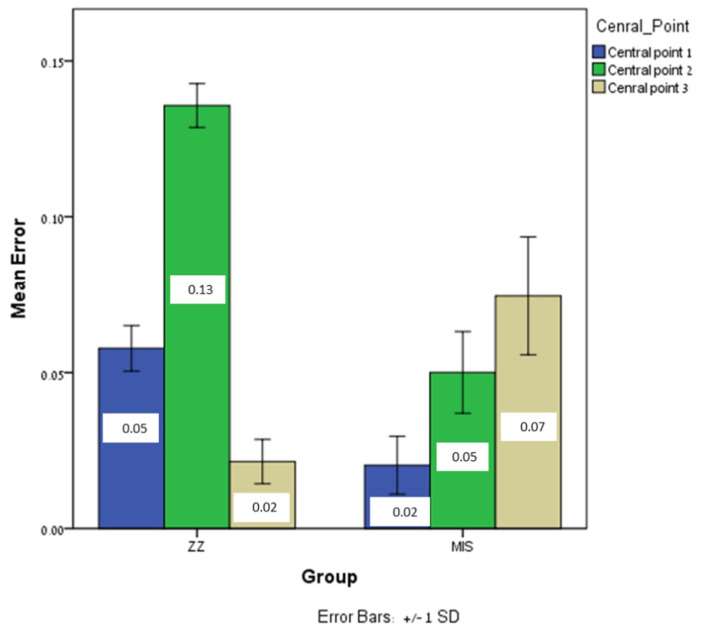
The mean intra-implant distance (central points 1, 2, 3) error and ±SD for ZZ and MIS.

Significant differences were found between MIS and ZZ regarding intra-implant distance: central point 1 (*p* = 0.0001), central point 2 (*p* = 0.0001), and central point 3 (*p* = 0.0001). The mean error for MIS was lower in central point 1 and 2 than that for ZZ, in contrast to central point 3, which was higher for MIS (Figure 6).

**Figure 7:**
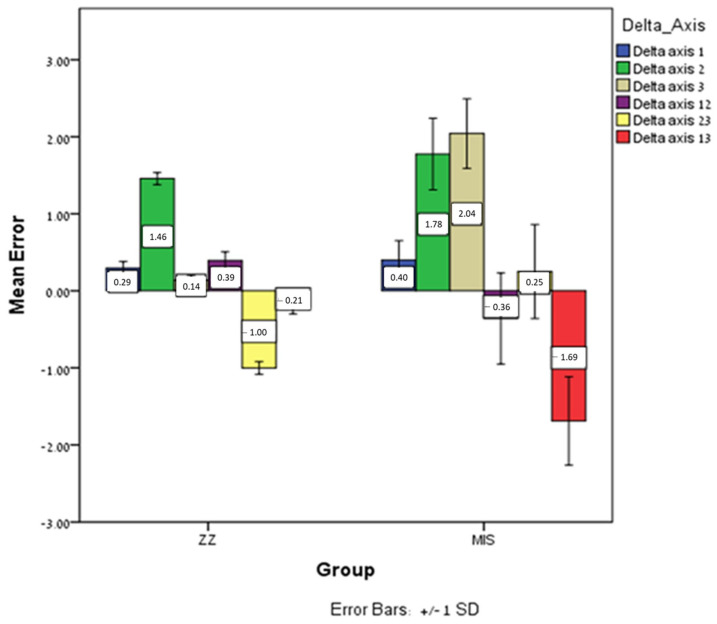
The mean intra-implant angle and inter-implant angle (delta axes 1, 2, 3 and delta-axes 12, 23, 13) error and ±SD for ZZ and MIS.

Significant differences were found for intra-implant angle (delta axis 1 (*p* = 0.05), delta axis 2 (*p* = 0.001), and delta axis 3 (*p* = 0.0005)) and for inter-implant angle (delta axes 12 (*p* = 0.0005), delta axes 23 (*p* = 0.0005), and delta axes 13 (*p* = 0.0005)). The mean error for MIS was lower in delta axes 12 and 23 than for ZZ, in contrast to that in delta axes 1, 2, 3, and 13, which was higher (Figure 7).

## 4. Discussion

In this pilot in vitro study, we suggest a method that can evaluate the differences (trueness) in several parameters (inter-implant distance, intra-implant distance, inter-implant angle, and intra-implant angle) between the LBS (gold standard) and IOS by using two different scan abutments.

When analyzing our results, we can conclude that when using two different ISBs, there are significant differences for inter-implant distance, intra-implant distance (central points), intra-implant angle, and inter-implant angle. This means that our null hypothesis must be rejected as there are significant differences between the LBS (gold standard) and IOS and between the two ISBs.

The results indicated that the inter-implant distance for the MIS was significantly lower than that for the ZZ. One of the main differences between the two ISBs is their geometry. The geometry of the MIS ISB was trapezoid with sharp angles and it a larger surface area compared to the ZZ ISB, which was cylindrical with no angles. We assume that because the MIS ISB had a larger area of the upper plane and side plane compared to the ZZ ISB and it was perpendicular to the occlusal surface (unlike the ZZ abutment where the side plane was not perpendicular but more continuous), this may affect the results as the inter-implant distance with the MIS ISB was shorter compared to the ZZ ISB. This means that the geometry of the ISB influenced the results. Several studies have already investigated the relationships between the inter-implant distance and scanning errors and, in agreement with our study, they showed that as the inter-implant distance increases, the distortion also increases (which decreases the trueness) [24,25,26].

Moreover, because of the special geometry of the MIS ISB, we have more options to reduce the inter-implant distance, which we cannot do with a cylindrical shape like the ZZ ISB. It is important to mention that in this study, we used a straight-line edentulous space, and we know from the literature that as the space enlarges (complete edentulous jaw) and we do not have good landmarks like teeth, the distortion increases [27,28]. The results indicate that the intra-implant angle for the ZZ ISB was significantly lower compared to that for the MIS ISB. We assume that because of the cylindrical shape of the ZZ ISB, it is easier to define and superimpose the axis. As for this point, in our previous study for a single ZZ ISB, it was found that one piece with a cylindrical shape had the lowest intra-implant angle (0.4 mm) compared to two other ISBs (MIS and AB) [29].

For the inter-implant angles, the results were mixed. In some of the cases, the inter-implant angle was significantly lower for the ZZ, and for the other cases, it was lower for the MIS. For the short distances (12, 23), the MIS had lower inter-implant angle changes, and for the long distance (13), the ZZ had lower inter-implant angle changes. These results are interesting as we expected that because the intra-implant angle was lower for the ZZ ISB compared to the MIS ISB, the inter-implant angle will be the same, but this is not the case between the distal and mesial implants.

It is already known from the literature that the geometry of the scan abutments, one-piece or two pieces, the materials, and the scanning techniques are some of the parameters that may affect the accuracy [15,30], In the current study, we decided to use only the titanium ISB as we realized in our previous research that PEEK is less accurate and we recommended the use of titanium [29]. Pan et al. tested two different scan abutments and their impact on accuracy. They used a laboratory scanner and scanned two different types of ISBs (dome-shaped and cuboidal), in an edentulous maxilla with six implants at the multi-unit level. Like our results, they showed that the geometry of the scan abutments has a direct impact on the accuracy [31].

Andriessen et al. reported that the intra-implant angle deviation must be limited to 0.194 degrees for an implant length of 14.8 mm, because 0.194 degrees may create 50 μm of difference at the apex of the implant. Hence, we want to achieve less than 0.4 degrees of inter-implant angle error for a physiologic bone strain. However, it is reported in the literature that the misfit can be in the range between 22 and 100 μm, so the number of 100 μm or less is not as strict for a passive fit [32,33,34].

In our study, we achieved changes for the inter-implant angle between 0.011 degrees and 0.131 degrees, which is under the 0.194 degrees that has been suggested by Andriessen.

Rutkunas et al. showed in their study that an angulation of more than 10 degrees between implants could negatively affect the passive fit when using fabricated restorations with a digital intra-oral scanner. In our study, the change in deviation in both intra-implant angle and inter-implant angle did not exceed more than 2.07 degrees [35].

Another issue worth mentioning is muscular fatigue when using the IOS. Scanning multiple times can affect the muscles and may lead to different results of the scanning ISB. This may affect the results and may be one of the limitations of this study [36].

The present pilot study has suggested a method to measure the inter-implant distance, intra-implant distance, inter-implant angle, and intra-implant angle between the laboratory scanner and intra-oral scanner by using two different ISBs for three implants in a straight line. There is still a lack of information in the literature regarding what is the threshold of the distance and angles between implants for which we may still receive an acceptable passive fit for prosthetic work on implants and choose the type of restoration between screw-retained and cemented.

This study has several limitations, including the in vitro design, the use of only one intra-oral scanner and one laboratory scanner, the superimposition being performed using a best-fit algorithm and not automatically, the use of only two ISBs, and successive scans being performed by one practitioner.

## 5. Conclusions

1. The geometry of the scan abutment has an impact on the inter-implant distance, inter-implant angle, intra-implant distance, and intra-implant angle.
2. The error for the inter-implant distance was significantly lower for the MIS ISB.
3. The error for the intra-implant distance was inconsistent between the ZZ ISB and MIS ISB.
4. The error for the inter-implant angle was inconsistent between the ZZ ISB and MIS ISB.
5. The error for the intra-implant angle was significantly lower for the ZZ ISB.

## Figures and Tables

**Table 1:** Mean error; ±SD; range; P25 (25th percentile), P50 (50th percentile), and P75 (75th percentile) of angle; and distance from first to middle implant, distance from middle to last implant, and distance from first to last implant (central points 1, 2, 3; delta axes 1, 2, 3, 12, 23, 13) of both ZZ and MIS.

	ZZ Scan Abutment					MIS Scan Abutment		
	Mean (±SD) Range	P25	P50	P75	Mean (±SD) Range	P25	P50	P75
Inter-implant Distance 12 (mm)	0.081 (±0.014) 0.074	0.072	0.081	0.086	−0.036 (±0.029) 0.155	−0.051	−0.039	−0.022
Inter-implant Distance 23 (mm)	−0.131 (±0.010) 0.044	−0.138	−0.133	−0.124	0.011 (±0.019) 0.088	0.000	0.013	0.021
Inter-implant Distance 13 (mm)	−0.045 (±0.011) 0.045	−0.052	−0.046	−0.038	−0.020 (±0.018) 0.077	−0.034	−0.020	−0.006
Intra-implant distance (Central point 1) (mm)	0.057 (±0.007) 0.023	0.049	0.057	0.064	0.020 (±0.009) 0.037	0.012	0.019	0.025
Intra-implant distance (Central point 2) (mm)	0.135 (±0.007) 0.032	0.132	0.135	0.140	0.050 (±0.013) 0.054	0.040	0.047	0.059
Intra-implant distance (Central point 3) (mm)	0.021 (±0.007) 0.030	0.016	0.021	0.027	0.074 (±0.018) 0.095	0.065	0.073	0.083
Intra-implant angle Delta axis 1 (angle)	0.294 (±0.084) 0.457	0.244	0.311	0.344	0.400 (±0.251) 1.211	0.245	0.355	0.495
Intra-implant angle Delta axis 2 (angle)	1.457 (±0.077) 0.335	1.410	1.463	1.498	1.776 (±0.464) 2.182	1.442	1.675	2.024
Intra-implant angle Delta axis 3 (angle)	0.139 (±0.059) 0.243	0.094	0.142	0.189	2.042 (±0.451) 2.301	1.832	1.975	2.256
Inter-implant angle Delta axis 12 (angle)	0.392 (±0.113) 0.551	0.300	0.407	0.469	−0.359 (±0.590) 2.650	−0.810	−0.511	−0.065
Inter-implant angle Delta axis 23 (angle)	−1.001 (±0.082) 0.331	−1.067	−0.995	−0.947	0.2499 (±0.610) 3.570	−0.111	0.250	0.540
Inter-implant angle Delta axis 13 (angle)	−0.209 (±0.089) 0.399	−0.266	−0.217	−0.157	−1.690 (±0.573) 2.921	−2.070	−1.724	−1.459

## Data Availability

The data presented in this study are available on request from the corresponding author.
